# Physical health attitude scale among mental health nurses in Taiwan: Validation and a cross-sectional study

**DOI:** 10.1016/j.heliyon.2023.e17446

**Published:** 2023-06-24

**Authors:** Wen-Chii Tzeng, Hsin-Pei Feng, Chia-Huei Lin, Yue-Cune Chang, Mark Haddad

**Affiliations:** a School of Nursing, National Defense Medical Center, Taipei, Taiwan; b Department of Nursing, Tri-Service General Hospital, Taipei, Taiwan; c Department of Mathematics, Tamkang University, New Taipei, Taiwan; d School of Health Sciences, City University of London, London, United Kingdom

**Keywords:** Attitude, Instrument adaptation, Mental health, Nursing education, Physical health care, Reliability, Validity

## Abstract

The Physical Health Attitude Scale (PHASe) is an internationally valid and reliable scale for assessing mental health nurses' attitudes toward providing physical health care to people with serious mental illness. This study translated the PHASe into traditional Chinese and evaluated its psychometric properties in the context of Taiwan. A descriptive, cross-sectional study design was adopted, and convenience sampling was used to recruit 520 mental health nurses from 11 hospitals across Taiwan. Data were collected between August and December 2019. Brislin's translation model was used for the validation process. Exploratory factor analysis and confirmatory factor analysis were used to establish the construct validity of the scale, and Cronbach's alpha and composite reliability were used to determine its reliability. The factor analysis results revealed that the 4-factor 17-item traditional Chinese version of the PHASe accounted for 44.2% of the total variance. Each factor had adequate internal consistency (Cronbach's alpha = 0.70 to 0.80). We also noted significant differences between groups with different attitudes, demonstrating known-group validity. Our findings indicate that the traditional Chinese version of the PHASe is acceptable for evaluating nurses' attitudes toward providing physical health care in Taiwan.

## Abbreviations

PHASe = Physical Health Attitude Scale
EFA = exploratory factor analysis
CR = composite reliability

## 1 Introduction

Globally, people with serious mental illness, such as schizophrenia, bipolar disorder, and major depressive disorder, have a higher risk of physical health problems, including cancer, metabolic disorders, cardiovascular disease, viral disease, and respiratory tract disease, than do people without such illness [1,2]. The mortality rate in people with serious mental illness has been reported to be higher than that in people without such illness [3,4]; moreover, the life expectancy of people with serious mental illness is approximately 15 years shorter than that of individuals without such illness [5]. In Taiwan, individuals with serious mental illness were reported to experience higher mortality rates and healthcare expenditure than do individuals without such illness [6], and this gap appears to be increasing [7]. People with a diagnosis of serious mental illness do not appear to be benefiting from advances made in health care. Because nurses play an essential role in the mental healthcare system, improving their ability to provide the required physical health care and implement early interventions is crucial. Although awareness of comorbid physical and mental diseases is growing, a valid scale for measuring mental health nurses’ attitudes toward physical health care has yet to be developed specifically for culturally Chinese societies.

Serious mental illness can be defined as a mental, behavioral, or emotional disorder that substantially interferes with an individual's life activities, such as their interpersonal relationships, daily living activities, self-care, employment, and recreation [8]. An accumulating body of evidence indicates that multiple factors can negatively influence the physical health of people with serious mental illness, including genetic predispositions; psychiatric symptoms; responsiveness to antipsychotic medication; and willingness to make lifestyle changes, such as tobacco cessation, regular physical activity, and healthy diet implementation [9]. These factors also interact with patients' disadvantaged social and economic status and with their psychosocial stressors and risk behaviors, including substance use and suicidal or violent behaviors. Therefore, nurses must have the necessary skills to motivate and support people with serious mental illness in order to improve their physical health.

Healthcare disparities across the care continuum that involves screening, diagnosis, treatment, and end-of-life care are influenced by the pervasive stigma of mental illness [10]. This stigma also affects the interactions between patients and health care professionals, who often not only consider people with serious mental illness to be difficult but also face challenges in diagnosing and treating medical diseases in such patients, particularly when the patients exhibit bizarre affect, violent behavior, or poor health literacy [11]. A study conducted in Taiwan reported that compared with individuals without schizophrenia, those with schizophrenia had a 1.3- to 1.8-fold increased risk of experiencing adverse clinical events, such as intensive care unit admission, acute respiratory failure, or mechanical ventilation use, during their medical and surgical hospitalizations [12]. Therefore, increasing awareness of health disparities and improving mental health nurses’ understanding of the barriers to physical health care for people with serious mental illness are essential.

Mental health nurses typically focus on providing care to improve patients' mental health; however, such nurses often perceive themselves to have inadequate physical health care knowledge and skills and lack confidence in delivering physical health care [13]. Gray and Brown [14] interviewed 18 Australian mental health nurses and 15 people with serious mental illness to obtain information about their experiences with working together to improve physical health; they determined that the nurses did not prioritize improving the patients' physical health, and the patients reported that their physical health needs were not adequately addressed. This finding indicates that the attitudes of mental health nurses are crucial because these attitudes may negatively influence nurses’ ability to detect and manage comorbid mental and medical diseases.

Price [15] defined attitude as an individual's tendency to react to certain situations on the basis of their beliefs and experiences. Attitude is a psychological construct that develops within an individual's mind and cannot always be clearly defined. However, attitude was reported to comprise three components: affection, cognition, and behavior (also known as the ABC model of attitude) [16]. Robson and Haddad [17] developed and validated a tool, namely the Physical Health Attitude Scale (PHASe), on the basis of the ABC model of attitude, for measuring mental health nurses' attitudes toward the provision of physical health care to people with serious mental illness. The psychometric properties of the PHASe have been tested in various languages, including Arabic [18], Japanese [19], and Turkish [20], and this has enabled international comparisons between the attitudes of nurses in various regions and countries, such as Australia, Canada, Hong Kong, Qatar, the United States, and the United Kingdom [21].

Considering the increasing prevalence of physical health problems and the increasing mortality rate in people with serious mental illness, developing a suitable tool for measuring nurses' ability to provide physical health care and to implement early interventions for people with serious mental illness is imperative. The validated PHASe can help nurse educators identify factors associated with mental health nurses’ attitudes toward the provision of physical health care; nevertheless, a Chinese version of the PHASe has not been developed. Accordingly, the current study presents a traditional Chinese version of the PHASe and determined its psychometric properties.

## 2 Materials and methods

**Design** This study applied a descriptive, cross-sectional study design and adhered to the recognized reporting guidelines encompassed in Strengthening the Reporting of Observational Studies in Epidemiology.

**Participants and settings** Mental health services in Taiwan are provided in both inpatient and community settings. In addition, general hospitals in the public sector are required to provide mental health services such as inpatient psychiatric care, rehabilitation care, day care, and home care. In the present study, convenience sampling was used to recruit eligible nurses from 11 hospitals across Taiwan, comprising 5 mental health hospitals (1 in northern Taiwan, 2 in central Taiwan, 1 in southern Taiwan, and 1 in eastern Taiwan), 5 regional hospitals (2 in northern Taiwan, 1 in central Taiwan, 1 in southern Taiwan, and 1 in eastern Taiwan), and 1 medical center in Taipei. The medical center has 200 psychiatric inpatient beds, and each of the 5 regional hospitals has more than 50 psychiatric inpatient beds. At the time of data collection, approximately 5500 nurses were working in mental health settings in Taiwan [22], and 835 of these nurses were working in mental health settings in the 11 selected hospitals.

The inclusion criteria were as follows: (1) being a full-time direct care registered nurse, (2) being aged 20 years or older, (c) having at least 1 year of psychiatric mental health nursing experience, and (d) being currently employed in one of the selected hospitals. Nurses who did not provide direct care to individuals with mental illness, including head nurses, supervisors, and directors of the nursing department, were excluded.

A general rule of thumb for factor analysis is that the minimum sample size should be 10 cases per item [23]. The original PHASe comprises 28 items; accordingly, the minimum sample size was considered to be 280. A total of 669 nurses met the eligibility criteria, and 620 expressed interest in participating in the study. Of the 620 nurses, 547 returned their questionnaires (88.2% return rate). Among the returned questionnaires, 27 were incomplete (2 nurses did not respond to any questionnaire items, and 25 did not complete the PHASe). Analysis of the data obtained from the complete and incomplete responses revealed no significant selection bias with respect to the demographic and job-related variables. After the 27 incomplete responses were removed, the valid response rate was 83.9% (*n* = 520; attrition rate = 16.1%). A post hoc power analysis (executed in G × Power version 3.1) indicated that a sample size of 520 respondents in a study involving 13 predictors and covariates, a medium effect size of 0.15 [24], and a significance level of 0.05 would yield a statistical power of >0.99.

**PHASe instrument** Data were collected using a demographic data form and the traditional Chinese version of the PHASe. The demographic data that were collected comprised age, gender, educational background, nursing experience, and other related demographic variables.

The PHASe is a 28-item instrument, and each item is scored on a 5-point Likert scale with endpoints ranging from 1 (*strongly disagree*) to 5 (*strongly agree*) [17]. The PHASe has 12 negatively worded items, and its total score ranges from 28 to 140. A higher score indicates a more positive attitude. The Cronbach's alpha value for the total scale was 0.76, and the Cronbach's alpha values for the subscales: attitude toward involvement in physical health care (10 items), confidence in delivering physical health care (6 items), perceived barriers to physical health care delivery (7 items), and attitude toward smoking (5 items) were 0.86, 0.74, 0.67, and 0.61, respectively [17].

Brislin's translation model was used for the translation and validation of the PHASe [25]. After authorization was obtained from the two original authors of the PHASe [17], two members of our research team who were proficient in English and Chinese and had experience in translating scholarly nursing-related writing translated the scale to Chinese. The first Chinese version was then back-translated to English by a professional translation company that had not participated in the first translation or seen the original English version. After the congruence of culturally equivalent meanings between the two versions was ensured, the final back-translated version was emailed to Dr Haddad, one of the original creators, to verify whether the back-translated version was identical to the original version. This process was repeated until the back-translated and original items conveyed the same meaning.

Five experts (two clinical nurses with a master's degree in nursing, one doctoral nursing student, and two academics in the field of nursing) with experience working with people with serious mental illness were invited to rate the cultural equivalence, relevance, and appropriateness of the traditional Chinese version of the PHASe by using a 4-point Likert scale with the anchors *strongly disagree, disagree, agree, and strongly agree*. Items with a mean score of ≥3.0 were retained [26]. The average item-level content validity index for all items was 0.98. No items were deleted from the traditional Chinese version of the scale. The final version was obtained after the elimination of redundant wording and adjectives on the basis of the panel's recommendations.

**Data collection** Data were collected between August and December 2019. After obtaining approval from the head of the nursing department of each hospital, a research assistant visited each hospital to meet with potential participants during their break time to introduce the purpose of the study. Eligible nurses received an information sheet outlining the study aims and the procedures that would be employed to ensure confidentiality. After providing written informed consent, the participants received an anonymous questionnaire and a stamped return envelope. All questionnaires were returned in sealed envelopes to the principal investigator by mail.

**Ethical considerations** This study was approved by the institutional review board of the principal investigator's medical center (Reference No. 1-108-05-092). All participants agreed to participate in the study after being given adequate time to make a decision, and written consent was obtained prior to data collection. Agreement to participate was voluntary and anonymous. The participants were informed that their decision to participate or withdraw during the study period (their data would no longer be collected upon their withdrawal) would have no bearing on their employment or working conditions at their respective hospitals.

**Analysis** Data were managed and analyzed using SPSS (version 20.0; SPSS, Chicago, IL, USA) and Amos (version 18; SPSS), with the level of significance set at 0.05. The demographic data are presented as *n* (%) for categorical variables and as means (standard deviations [SDs]) for continuous variables.

Construct validity was determined using exploratory factor analysis (EFA) and known-group validity. Specifically, EFA was conducted using the maximum likelihood method and oblique rotation to explore the factor structure of the traditional Chinese version of the PHASe [27]. Factors were retained if they had eigenvalues of >1.0—indicating that a factor explained a higher proportion of the total variance than did a single item included in the analysis—and were assessed using a scree plot. Items with factor loadings of <0.5 or with cross-loading were excluded [28].

Known-group validity was used to determine whether the traditional Chinese version of the PHASe could discriminate between groups with different attitudes [26]. On the basis of the findings of Robson et al. [29], nurses of the male gender, with a nonsmoking status, with a bachelor's degree or higher education level, with adult care experience, and with physical health care training were hypothesized to have higher total and subscale PHASe scores. The mean total and subscale scores derived for the predefined groups were compared using independent *t* tests, and the effect size (Cohen's d) was calculated as the mean difference between two groups divided by the pooled standard deviation, which indicated the magnitude of the difference between the groups. Cohen [30] classified effect sizes as follows: small effect (d = 0.2), moderate effect (d = 0.5), and large effect (d = 0.8). Internal consistency was assessed using the Cronbach's alpha coefficient. A Cronbach's alpha value of ≥0.7 was considered to indicate acceptable reliability [26]. Composite reliability (CR) was used to measure the internal consistency of a set of items within a latent construct. A CR of ≥0.7 indicated that items belonging to the same factor were highly correlated and that no items were unreliable [23].

## 3 Results

### 3.1 Participants

Table 1 presents the demographic characteristics of the participants. The mean age of the participants was 36.7 (SD = 8.95) years. The majority of the participants were female (91.7%), were working in mental health hospitals (66.7%), and had at least a bachelor's degree in nursing (64.0%). Moreover, the participants had 1–32 years of experience as mental health nurses, with the mean years of experience being 9.23 years. Although 50.4% (*n* = 262) of the participants had adult care experience, only 74 (14.2%) had attended in-service physical health care training within the 5 years preceding the study.

**Table 1:** Participant demographics (*n* = 520).

Variable	n	%	Mean	SD	Range
Age (years old)			36.70	8.95	21–62
<40	317	61.0			
≥40	203	39.0			
Gender					
Men	43	8.3			
Women	477	91.7			
Level of education					
Associate degree	187	36.0			
≥ Bachelor of Science degree	333	64.0			
Smoking					
Current/formerly	15	2.9			
Never	505	97.1			
Hospital type					
Mental health hospital	347	66.7			
General hospital	173	33.3			
Work unit					
Acute psychiatric ward	304	58.5			
	216	41.5			
Nursing background					
Years of mental health nursing practice			9.23	6.93	1–32
<15	403	77.5			
≥15	117	22.5			
Adult care experience					
Yes	262	50.4			
No	258	49.6			
Attended physical health care training programme					
Yes	74	14.2			
No	446	85.8			

### 3.2 EFA results

EFA was conducted using the maximum likelihood method and oblique rotation. The initial analysis yielded seven factors that accounted for 41.57% of the total variance; however, seven items (4, 5, 11, 13, 23, 27, and 28) were excluded because their factor loadings were <0.5, and one item (20) was excluded because it exhibited cross-loading. Subsequently, EFA was conducted on the remaining 20 items. The analysis yielded five factors that accounted for 42.99% of the total variance; nevertheless, three items (8, 14, and 24) were also removed because they had factor loadings of <0.5. For the remaining 17 items, 2 factors (2 and 5) did not contain at least 3 items. A comparison of factor models with three- and four-factor structures was performed. All six items of the subscale ‘confidence in delivering physical health care’ of the original PHASe [17] were excluded because their factor loadings were <0.5 in the model with the three-factor structure; however, in the model with the four-factor structure, the remaining 17 items were loaded onto the factors, similar to the original PHASe (Table 2). Factor 1 (involvement in physical health care; six items) accounted for 19.8% of the total variance. Factor 2 (attitude toward smoking; four items) accounted for 10.4% of the total variance. Factor 3 (perceived obstacles to engagement in physical health care; three items) accounted for 8.2% of the total variance. Finally, factor 4 (confidence in physical health care practice; six items) accounted for 5.7% of the total variance.

**Table 2:** Factor loadings of the 17-item Chinese version of the PHASe.

No.	Statement	Factor 1^a^	Factor 2^a^	Factor 3^a^	Factor 4^a^
22	Ensuring that patients have their eyes regularly assessed by an optician should be part of the mental health nurses' role	0.745			
10	Ensuring that patients are registered with a dentist should be part of the mental health nurses' role	0.736			
25	Mental health nurse should educate male patients on the importance of testicular self-examination	0.681			
17	Mental health nurses should educate female patients on the importance of breast self-examination	0.646			
7	Verifying that patients have had cancer screenings (i.e. cervical smear/mammogram) should not be part of the mental health nurse's role.	0.583			
6	Advising on heart disease prevention should be part of the mental health nurses' role	0.538			
20	Patients should be banned from smoking on all Healthcare premises		0.886		
16	Patients should be given cigarettes to help achieve therapeutic goals		0.624		
14	Staff should be banned from smoking on all Healthcare premises		0.579		
12	Patients should not be encouraged to give up smoking, because they have enough to cope with		0.534		
18	Encouraging patients to follow healthy-eating advice is difficult			0.780	
9	It is difficult to get clients to follow advice on how to manage their weight			0.714	
15	Patients are not motivated to exercise			0.532	
19	I am confident in assessing signs and symptoms of hypoglycemia				0.701
3	I am confident in assessing signs and symptoms of hyperglycemia				0.663
26	I am confident that I could resuscitate a patient with cardiac arrest				0.543
21	I am confident that I know which psychotropic drugs increase the risk of cardiac problems				0.543
Eigenvalue		4.174	2.067	1.926	1.449
Variance explained (%)		19.8%	10.3%	8.2%	5.7%
Cronbach's alpha		0.81	0.75	0.70	0.70
Composite reliability		0.80	0.74	0.72	0.70

a Factor 1 = Involvement in physical healthcare, Factor 2 = Attitude to smoking, Factor 3 = Perception of obstacles to engagement in physical healthcare, and Factor 4 = Confidence in physical healthcare practice.

### 3.3 Reliability

The Cronbach's alpha coefficient was used to assess the internal consistency reliability of the total scale and its subscales. The Cronbach's alpha coefficient derived for the 17-item traditional Chinese version of the PHASe was 0.80, and the Cronbach's alpha coefficient derived for subscales ‘involvement in physical health care,’ ‘attitude toward smoking,’ ‘perceived obstacles to engagement in physical health care,’ and ‘confidence in physical health care practice’ were 0.82, 0.75, 0.70, and 0.70, respectively (Table 2). The CR values derived for the four subscales ranged from 0.70 to 0.80, indicating that items belonging to the same subscales were correlated.

### 3.4 Known-group validity

As presented in Table 3, the mean total PHASe scores were significantly higher among participants with a nonsmoking status (*t* = 2.05, *p* = 0.041), adult care experience (*t* = 2.23, *p* = 0.026), and physical healthcare training (*t* = 2.51, *p* = 0.012) than they were among the other participants. The mean score derived for ‘involvement in physical health care’ was significantly higher in participants with a nonsmoking status than those with smoking status (t = 2.07, *p* = 0.038). Furthermore, the mean score derived for ‘attitude toward smoking’ differed with respect to gender (t = −2.61, *p* = 0.009) and adult care experience (t = 3.55, *p* < 0.001). The mean score derived for ‘confidence in physical health care practice’ was positively associated with adult care experience (t = 5.30, *p* < 0.001) and physical health care training (t = 3.40, *p* = 0.001). No group differences were observed in the mean score derived for ‘perceived obstacles to engagement in physical health care.’

**Table 3:** Comparison of scores overall and for each of the four subscales of the Chinese version of the PHASe by demographics (*n* = 520).

Variable	Factor 1^a^			Factor 2^a^			Factor 3^a^			Factor 4^a^			Total Score		
	Mean (SD)	*P*	ES^b^	Mean (SD)	*P*	ES^b^	Mean (SD)	*P*	ES^b^	Mean (SD)	*P*	ES^b^	Mean (SD)	*P*	ES^b^
Gender															
Men (*n* = 43)	19.56 (4.171)	0.476	0.11	15.07 (3.487)	**0.009**	0.38	6.47 (2.164)	0.081	0.28	15.33 (1.899)	0.749	0.05	56.42 (8.438)	0.218	0.18
Women (*n* = 477)	19.09 (4.162)			16.27 (2.837)			7.08 (2.196)			15.42 (1.879)			57.86 (7.210)		
Level of education															
Associate degree (*n* = 187)	18.65 (3.875)	0.052	0.18	15.85 (2.778)	0.058	0.17	7.02 (2.130)	0.978	0.00	15.41 (1.982)	0.988	0.00	56.94 (6.645)	0.052	0.17
≥ BS^c^ degree (*n* = 333)	19.39 (4.295)			16.35 (2.972)			7.03 (2.238)			15.41 (1.684)			58.19 (7.647)		
Smoking															
Currently/formerly (*n* = 15)	16.93 (4.061)	**0.038**	0.55	15.07 (3.955)	0.286	0.33	6.73 (1.831)	0.602	0.15	15.20 (1.781)	0.656	0.12	53.93 (8.102)	**0.041**	0.51
Never (*n* = 505)	19.19 (4.149)			16.21 (2.873)			7.03 (2.209)			15.42 (1.883)			57.85 (7.274)		
Adult care experience															
Yes (*n* = 262)	18.92 (4.122)	0.267	0.10	16.62 (2.465)	<**0.001**	0.31	7.06 (2.158)	0.677	0.04	15.84 (1.764)	<**0.001**	0.47	58.44 (6.945)	**0.026**	0.20
No (*n* = 258)	19.33 (4.197)			15.72 (3.246)			6.98 (2.240)			14.98 (1.898)			57.02 (7.630)		
Prior physical healthcare training															
Yes (*n* = 74)	19.91 (4.317)	0.081	0.22	16.53 (2.873)	0.259	0.14	7.18 (2.278)	0.525	0.08	16.09 (1.830)	**0.001**	0.43	59.70 (7.851)	**0.012**	0.31
No (*n* = 446)	19.00 (4.124)			16.11 (2.917)			7.00 (2.186)			15.30 (1.865)			57.41 (7.186)		

a Factor 1 = Involvement in physical healthcare (six items), Factor 2 = Attitude to smoking (four items), Factor 3 = Perception of obstacles to engagement in physical healthcare (three items), and Factor 4 = Confidence in physical healthcare practice (six items).

b ES = effect size.

c BS = Bachelor of Science.

The effect sizes of the relationships of smoking with total score and ‘involvement in physical health care’ were moderate (0.51 and 0.55, respectively). The effect size of the relationship between gender and ‘attitude toward smoking’ was low (0.38). Moreover, the effect sizes of the relationships of adult care experience with total score, ‘attitude toward smoking,’ and ‘confidence in physical health care practice’ were low (0.20, 0.31, and 0.47, respectively). The effect sizes of the relationships of prior physical health care training with total score and ‘confidence in physical health care practice’ were low (0.31 and 0.43, respectively).

## 4 Discussion

In the present study, we translated the PHASe into traditional Chinese and then evaluated its psychometric properties in the context of Taiwan by including a sample of 520 mental health nurses. Our EFA results reveal four factors with eigenvalues of >1 and factor loadings of >0.5; the remaining 17 items were loaded on the corresponding factors of the original PHASe. Each factor also had adequate internal consistency reliability.

Our EFA results confirm the validity of the four-factor structure of the traditional Chinese version of the PHASe, which is similar to the structure of the original instrument. However, 11 items were removed from the traditional Chinese version of the PHASe. The original version of the PHASe, which was developed in the United Kingdom, comprises 28 items, whereas the Canadian, Turkish, and Chinese versions comprise 15, 24, and 17 items, respectively. Siren et al. [31] developed the two-factor Canadian version of the PHASe; they combined the involvement and perceived barrier components but removed the smoking component. Ozaslan et al. [20] developed the four-factor Turkish version of the PHASe; however, in their model, some items were loaded on different factors, and the name of factor 4 was changed, namely attitudes toward smoking and negative beliefs, because two domains were combined for the factor. These differences may have occurred because mental health nurses from different cultures have different perspectives on their roles in the process of providing physical health care.

The four-factor model derived in the present study explained a relatively low proportion of the total variance (44.2%); however, this proportion is consistent with the proportions of the variance explained by the original (42.1%) [17], validated Canadian (46.8%) [31], and Turkish (51.3%) [20] versions of the PHASe. A possible reason for the low total variance in the traditional Chinese version of the PHASe is that mental health nurses constitute a somewhat homogeneous group, which may have limited the range of EFA structures [32]. Another possible reason for the low total variance is that mental health nurses in Taiwan implement nursing care in accordance with national hospital accreditation standards, leading to consistency in the provision of physical health care in Taiwan.

We observed that in our version of the PHASe, ‘involvement in physical health care’ accounted for the highest proportion (19.8%) of the total variance. This factor also accounted for the highest proportion of the variance in the original (15.5%) [17] and Turkish (23.1%) [20] versions of the PHASe. However, we removed item 1 (“Helping patients control their weight is a responsibility of psychiatric nurses”), item 2 (“Psychiatric nurses should be responsible for providing nutritional advice to patients”), item 4 (“Psychiatric nurses should not provide exercise-related advice to patients”), and item 11 (“Psychiatric nurses should provide contraception advice to patients”) from the aforementioned factor in the traditional Chinese version of the PHASe because of their low factor loadings (<0.5), as revealed by our EFA. These items also had similar factor loadings in the Canadian version of the PHASe [31]. In the Turkish version, item 1 exhibited cross-loading on both ‘involvement in physical health care’ and ‘confidence in physical health care practice,’ and item 4 was removed from this version of the PHASe [20]. Chee et al. [24] reported that either item did not differ between generalist prepared nurses and mental health nurses in Australia. A possible reason for this finding is that the PHASe was developed before the World Health Organization published global recommendations regarding the minimum frequency, duration, intensity, type, and total amount of physical activity required for health [33]. Nurses have become increasingly aware of the importance of physical activity for noncommunicable disease prevention over the past decade. Therefore, the four items that were removed from the traditional Chinese version of the PHASe may not have sufficient discriminatory power to evaluate nurses' attitudes toward physical health care, despite them being crucial contributors to attitudes toward phyiscal health care. This finding requires further investigation in future studies.

Our results demonstrate that factor 2 (attitudes toward smoking) is similar to factor 4 of the original PHASe. Taiwan implemented the Tobacco Hazards Prevention Act in 2009 [34]. Since then, all hospitals have become smoke-free environments, and all hospital staff members are required to support the tobacco-free policy and to receive training on providing smoking-cessation services. The majority of the nurses who participated in our study reported not using tobacco in any form. However, a study conducted in Taiwan revealed that mental health nurses often have low self-efficacy and practical experience in providing smoking-cessation services for people with serious mental illness [35]. Therefore, we retained four of the five items to assess the nurses’ attitudes toward patient smoking cessation.

When developing the traditional Chinese version of the PHASe, we removed four items, namely item 5 (“Patients with severe psychiatric problems are uninterested in improving their physical health”), item 13 (“Informing patients of the potential effects of medications on their physical health can increase their noncompliance”), item 23 (“My workload prevents me from implementing any health-promotion measures”), and item 27 (“Patients are worried about their physical health mostly because of their mental illness”) from ‘perceived obstacles to engagement in physical health care’. Similarly, items 13, 23, and 27 were removed from the Canadian version of the PHASe [31]. In addition, in the Turkish version, items 13 and 27 were both loaded on attitudes toward smoking and negative beliefs [20]. These results should be expected because technology plays a crucial role in health education for people with serious mental illness. Furthermore, mental health nurses in Taiwan frequently use health information systems to perform and document health education that is provided in accordance with patients' health needs [36].

We assessed the known-group validity of the traditional Chinese version of the PHASe. The results reveal that gender, smoking status, education level, adult care experience, and physical health care training had moderate to small effect sizes, indicating that the traditional Chinese version of the PHASe and its subscales could discriminate between groups defined using the characteristics affecting mental health nurses' attitudes. However, we observed no significant differences in the mean scores for ‘perceived obstacles to engagement in physical health care’ between nurses with different personal and nursing backgrounds. A possible explanation for this finding is that all items in this domain were negatively phrased, which could have caused confusion in the participants because one negative phrase can have two inferred meanings in Chinese [37]. Another potential reason is that the training of mental health nurses in Taiwan is conducted using a system similar to those used in the United States and Australia; that is, nurses must obtain registered nurse qualifications before specializing in mental health. By contrast, in the United Kingdom, nurses begin their undergraduate programs by specializing in mental health and obtain registered psychiatric nurse qualifications. Compared with the participants in the study by Robson et al. [29], those in our study who had adult nursing care experience were more likely to perceive themselves as having physical health care knowledge and skills.

Regarding reliability, the 17-item traditional Chinese version of the PHASe had satisfactory internal consistency. The Cronbach's alpha value of the entire scale was 0.80, which is similar to those of the original (0.77) [17] and the Turkish (0.83) [20] versions of the PHASe. The reliability of the four subscales ranged from 0.70 to 0.80, which is comparable to the reliability obtained for samples of mental health nurses in the United Kingdom (0.61–0.86) [17], Australia (0.60–0.83) [24], Hong Kong (0.66–0.85), Japan (0.65–0.76), Qatar (0.36–0.77) [19], and Turkey (0.64–0.88) [20]. These findings reveal that the traditional Chinese version of the PHASe reliably assesses mental health nurses' attitudes toward physical health care.

The strength of this study is that the traditional Chinese version of the PHASe was verified using a sufficiently large sample of mental health nurses from diverse nursing backgrounds. Therefore, the traditional Chinese version of the PHASe with a four-factor structure may be useful for future studies of culturally Chinese societies. Moreover, the traditional Chinese version of the PHASe comprises only 17 items; hence, it can practically be used to measure mental health nurses’ attitudes toward physical health care.

Despite its strength, our study has some limitations. Specifically, we observed that the total variance in the four-factor structure was low. Therefore, a model with different factors could be developed to improve upon the variability explained by the present model. It is recommended to assess factor structure of the traditional version of the PHASe using confirmatory factor analysis on other samples in future studies. The traditional Chinese version of the PHASe should also be further investigated using multiple analyses to test its convergent validity, criterion validity, and test–retest reliability. Another limitation of this study is the limited generalizability of the findings. Although we used multisite sampling to obtain an adequate sample size, our results cannot be generalized to all mental health nurses because we recruited only nurses working in hospitals. Most of the participants were female and worked in acute inpatient psychiatric wards, which also limits the generalizability of our results. Additional cross-validated studies should be conducted using a diverse sample of nurses to confirm the four-factor structure of this traditional Chinese version of the PHASe. In addition, the data of the present study were obtained using self-report methods; thus, bias may be present in the results. Future research should clarify the psychometric properties of the traditional Chinese version developed in the current study.

## 5 Conclusions

The present study translated the PHASe into traditional Chinese and validated it by theoretically and empirically testing mental health nurses’ attitudes toward the provision of physical health care for people with serious mental illness in Taiwan. The reliability and validity of the 4-factor, 17-item traditional Chinese version of the PHASe are comparable to those of the original version of this scale. The traditional Chinese version of the PHASe could thus be used to evaluate the attitudes of mental health nurses toward physical health care within culturally Chinese societies and in cross-cultural studies. Furthermore, the findings of this study could serve as a reference for nurse educators when developing physical health care training programs that can help mental health nurses improve the management of comorbid mental and chronic diseases in patients.

## Author contribution statement

Wen-Chii Tzeng; Hsin-Pei Feng; Chia-Huei Lin: Conceived and designed the experiments; Performed the experiments; Analyzed and interpreted the data; Wrote the paper.

Yue-Cune Chang: Analyzed and interpreted the data; Contributed reagents, materials, analysis tools or data; Wrote the paper.

Mark Haddad: Conceived and designed the experiments; Wrote the paper.

## Funding statement

This study was supported by grants from the 10.13039/100020595National Science and Technology Council, Taiwan (grant number: MOST-108-2314-B-016-055) and Ministry of National Defense Medical Affairs Bureau (grant number: MND-MAB-D-112092).

## Data availability statement

Data will be made available on request.

## Declaration of competing interest

The authors declare that they have no known competing financial interests or personal relationships that could have appeared to influence the work reported in this paper.

## References

[1] Lambert A.M., Parretti H.M., Pearce E., Price M.J., Riley M., Ryan R., Tyldesley-Marshall N., Avşar T.S., Matthewman G., Lee A., Ahmed K., Odland M.L., Correll C.U., Solmi M., Marshall T. (2022). Temporal trends in associations between severe mental illness and risk of cardiovascular disease: a systematic review and meta-analysis. PLoS Med..

[2] Daré L.O., Bruand P.-E., Gérard D., Marin B., Lameyre V., Boumédiène F., Preux P.-M. (2019). Co-morbidities of mental disorders and chronic physical diseases in developing and emerging countries: a meta-analysis. BMC Publ. Health.

[3] Vai B., Mazza M.G., Delli Colli C., Foiselle M., Allen B., Benedetti F., Borsini A., Casanova Dias M., Tamouza R., Leboyer M., Benros M.E., Branchi I., Fusar-Poli P., De Picker L.J. (2021). Mental disorders and risk of COVID-19-related mortality, hospitalisation, and intensive care unit admission: a systematic review and meta-analysis. Lancet Psychiatr..

[4] Walker E.R., McGee R.E., Druss B.G. (2015). Mortality in mental disorders and global disease burden implications: a systematic review and meta-analysis. JAMA Psychiatr..

[5] Hjorthoj C., Sturup A.E., McGrath J.J., Nordentoft M. (2017). Years of potential life lost and life expectancy in schizophrenia: a systematic review and meta-analysis. Lancet Psychiatr..

[6] Wang J.Y., Chang C.C., Lee M.C., Li Y.J. (2020). Identification of psychiatric patients with high mortality and low medical utilization: a population-based propensity score-matched analysis. BMC Health Serv. Res..

[7] Pan Y.J., Yeh L.L., Chan H.Y., Chang C.K. (2020). Excess mortality and shortened life expectancy in people with major mental illnesses in Taiwan. Epidemiol. Psychiatr. Sci..

[8] National Institute of Mental Health USA (2017). https://www.nimh.nih.gov/health/statistics/mental-illness.shtml/.

[9] de Mooij L.D., Kikkert M., Theunissen J., Beekman A.T.F., de Haan L., Duurkoop P., Van H.L., Dekker J.J.M. (2019). Dying too soon: excess mortality in severe mental illness. Front. Psychiatr..

[10] Major B., Dovidio J.F., Link B.G. (2018).

[11] Aebi N.J., Caviezel S., Schaefert R., Meinlschmidt G., Schwenkglenks M., Fink G., Riedo L., Leyhe T., Wyss K. (2021). A qualitative study to investigate Swiss hospital personnel's perceived importance of and experiences with patient's mental-somatic multimorbidities. BMC Psychiatr..

[12] Chen Y.H., Lin H.C., Lin H.C. (2011). Poor clinical outcomes among pneumonia patients with schizophrenia. Schizophr. Bull..

[13] Happell B., Platania-Phung C., Scott D. (2014). What determines whether nurses provide physical health care to consumers with serious mental illness?. Arch. Psychiatr. Nurs..

[14] Gray R., Brown E. (2017). What does mental health nursing contribute to improving the physical health of service users with severe mental illness? A thematic analysis. Int. J. Ment. Health Nurs..

[15] Price B. (2015). Understanding attitudes and their effects on nursing practice. Nurs. Stand..

[16] Eagly A.H., Chaiken S. (1993).

[17] Robson D., Haddad M. (2012). Mental health nurses' attitudes towards the physical health care of people with severe and enduring mental illness: the development of a measurement tool. Int. J. Nurs. Stud..

[18] Ganiah A.N., Al-Hussami M., Alhadidi M.M.B. (2017). Mental health nurses attitudes and practice toward physical health care in Jordan, Community Ment. Health J.

[19] Bressington D., Badnapurkar A., Inoue S., Ma H.Y., Chien W.T., Nelson D., Gray R. (2018). Physical health care for people with severe mental illness: the attitudes, practices, and training needs of nurses in three Asian countries. Int. J. Environ. Res. Publ. Health.

[20] Ozaslan Z., Bilgin H., Uysal Yalcin S., Haddad M. (2020). Initial psychometric evaluation of the physical health attitude scale and a survey of mental health nurses. J. Psychiatr. Ment. Health Nurs..

[21] Dickens G.L., Ion R., Waters C., Atlantis E., Everett B. (2019). Mental health nurses' attitudes, experience, and knowledge regarding routine physical healthcare: systematic, integrative review of studies involving 7,549 nurses working in mental health settings. BMC Nurs..

[22] Ministry of Health and Welfare Taiwan (2020). https://dep.mohw.gov.tw/DOS/lp-1728-113.html/.

[23] Hair J.F. (2010).

[24] Chee G.L., Wynaden D., Heslop K. (2018). The provision of physical health care by nurses to young people with first episode psychosis: a cross-sectional study. J. Psychiatr. Ment. Health Nurs..

[25] Jones P.S., Lee J.W., Phillips L.R., Zhang X.E., Jaceldo K.B. (2001). An adaptation of Brislin's translation model for cross-cultural research. Nurs. Res..

[26] Polit D.F., Beck C.T. (2018).

[27] Nunnally J.C., Bernstein I.H. (1994).

[28] DeVellis R.F. (2017).

[29] Robson D., Haddad M., Gray R., Gournay K. (2013). Mental health nursing and physical health care: a cross-sectional study of nurses' attitudes, practice, and perceived training needs for the physical health care of people with severe mental illness. Int. J. Ment. Health Nurs..

[30] Cohen J. (1988).

[31] Siren A., Cleverley K., Strudwick G., Brennenstuhl S. (2018). Modification and initial psychometric evaluation of the physical health attitude scale for use in the Canadian mental health and addictions context, Issues Ment. Health Nurs..

[32] Fabrigar L.R., Wegener D.T., MacCallum R.C., Strahan E.J. (1999). Evaluating the use of exploratory factor analysis in psychological research, Psychol. Methods.

[33] World Health Organization (2020). https://www.who.int/publications/i/item/9789240015128/.

[34] Health Promotion Administration Taiwan (2020).

[35] Guo S.-E., Wang A.-L., Shu B.-C. (2015). Self-efficacy in providing smoking-cessation services among psychiatric nurses in central and southern Taiwan: an exploratory study. Int. J. Ment. Health Nurs..

[36] Dunn Lopez K., Gephart S.M., Raszewski R., Sousa V., Shehorn L.E., Abraham J. (2017). Integrative review of clinical decision support for registered nurses in acute care settings. J. Am. Med. Inf. Assoc..

[37] Chen I.H., Huang C.-R., Politzer-Ahles S. (2018). Determining the types of contrasts: the influences of prosody on pragmatic inferences. Front. Psychol..

