# Supplementary material for: Physical health attitude scale among mental health nurses in Taiwan: Validation and a cross-sectional study
Source: Heliyon. 2023 Jun 24;9(6):e17446. doi: 10.1016/j.heliyon.2023.e17446 (PMC10320265; doi:10.1016/j.heliyon.2023.e17446)
Supplement: Multimedia component 1 [file mmc1.docx]

Physical Health Attitude Scale

中文版身體健康照護態度問卷

Instruction: Please read the statement and rate the item that relates best to your personal opinion.

請閱讀下列敘述，並且在與您看法上最為相似的格子內打勾

|  |  | Strongly disagree  非常 不同意 | Disagree  不同意 | Uncertain  不確定 | Agree  同意 | Strongly agree  非常 同意 |
| --- | --- | --- | --- | --- | --- | --- |
| 1 | Helping clients manage their weight should be part of the mental health nurses role  幫助病人控制體重應該是精神科護理師角色之一部分 |  |  |  |  |  |
| 2 | Giving nutritional advice to clients should be part of a mental health nurses role  提供病人營養建議應該是精神科護理師角色之一部分 |  |  |  |  |  |
| 3 | I am confident in assessing signs and symptoms of hyperglycaemia  我對評估高血糖的徵象和症狀有信心 |  |  |  |  |  |
| 4 | It should not be the role of the mental health nurse to provide advice about exercise to clients  精神科護理師不應該提供病人有關運動的建議 |  |  |  |  |  |
| 5 | Clients with serious mental health problems are not interested in improving their physical health  有嚴重精神問題的病人對改善自己的身體健康並不感興趣 |  |  |  |  |  |
| 6 | Giving advice on how to prevent heart disease should be part of the mental health nurses role  提供如何預防心臟病的建議應該是精神科護理師角色之一部分 |  |  |  |  |  |
| 7 | It should not be the mental health nurse role to check with a client if they have had cancer screening checks (ie cervical smear/mammogram)  和病人確認是否已進行癌症篩檢不應該是精神科護理師角色之一部分 |  |  |  |  |  |
| 8 | I am confident that I can measure a clients blood-pressure accurately  我相信我能準確地為病人測量血壓 |  |  |  |  |  |
| 9 | It is difficult to get clients to follow advice on how to manage their weight  讓病人遵循控制體重的建議是很困難的 |  |  |  |  |  |
| 10 | Ensuring clients are registered with a dentist should be part of the mental health nurses role  確認病人定期去看牙醫是精神科護理師角色之一部分 |  |  |  |  |  |
| 11 | Mental health nurses should provide clients with contraceptive advice  精神科護理師應該提供病人避孕的建議 |  |  |  |  |  |
| 12 | Clients should not be encouraged to give up smoking, as they have enough to cope with  因為病人已經有太多問題無法應付，不應再鼓勵他們戒菸 |  |  |  |  |  |
| 13 | Informing clients about the possible effects medication may have on their physical health will increase non-adherence  告訴病人藥物會造成他們身體健康的影響有可能會增加他們的不遵從性 |  |  |  |  |  |
| 14 | Staff should be banned from smoking on all Healthcare premises  應該禁止工作人員在所有醫療機構內吸菸 |  |  |  |  |  |
| 15 | Clients are not motivated to exercise  病人沒有動機去運動 |  |  |  |  |  |
| 16 | Clients should be given cigarettes to help achieve therapeutic goals  應該讓病人獲得香菸來達到治療目標 |  |  |  |  |  |
| 17 | Mental health nurses should educate female clients about the importance of breast self-examination  精神科護理師應該衛教女性病人有關乳房自我檢查的重要性 |  |  |  |  |  |
| 18 | It is difficult to get clients to follow healthy-eating advice  很難讓病人遵循健康飲食的建議 |  |  |  |  |  |
| 19 | I am confident in assessing signs and symptoms of hypoglycaemia  我有信心評估低血糖的徵象和症狀 |  |  |  |  |  |
| 20 | Clients should be banned from smoking on all Healthcare premises  應該禁止病人在所有醫療機構中吸菸 |  |  |  |  |  |
| 21 | I am confident that I know which psychotropic drugs increase the risk that a client may experience cardiac problems  我相信自己知道哪些精神科藥物會增加病人出現心臟病的風險 |  |  |  |  |  |
| 22 | Ensuring clients have their eyes regularly checked by an optician should be part of the mental health nurses role  確保病人定期接受驗光師檢查眼睛應該是精神科護理師角色之一部分 |  |  |  |  |  |
| 23 | My workload prevents me doing any physical health promotion with clients  我的工作量使我無法為病人進行任何健康促進 |  |  |  |  |  |
| 24 | I am confident that I know which psychotropic drugs may cause damage to the eyes  我相信自己知道哪些精神科藥物會對病人眼睛造成傷害 |  |  |  |  |  |
| 25 | Mental health nurse should educate male clients about the importance of testicular self examination  精神科護理師應該衛教男性病人有關睪丸自我檢查的重要性 |  |  |  |  |  |
| 26 | I am confident that I could resuscitate a client who had a cardiac arrest  我相信我有能力急救心跳停止的病人 |  |  |  |  |  |
| 27 | Clients’ physical health worries are mostly due to their mental illness  病人擔心他們的身體健康大部份是因為他們的精神疾病所造成的 |  |  |  |  |  |
| 28 | Staff and clients smoking together helps to build a therapeutic relationship  工作人員和病人一起吸菸有助於建立治療性關係 |  |  |  |  |  |
